# Adaptation and illness severity: the significance of suffering

**DOI:** 10.1007/s11019-023-10155-x

**Published:** 2023-05-13

**Authors:** Borgar Jølstad

**Affiliations:** 1 The Health Services Research Unit—HØKH, Akershus University Hospital (Ahus), Sykehusveien 25, Akershus Universitetssykehus HF, Postboks 1000, 1478 Lørenskog, Norway; 2 Institute of Health and Society, Faculty of Medicine, University of Oslo, Oslo, Norway

**Keywords:** Health care priority setting, Well-being, Consequentialism, Adaptation

## Abstract

Adaptation to illness, and its relevance for distribution in health care, has been the subject of vigorous debate. In this paper I examine an aspect of this discussion that seems so far to have been overlooked: that some illnesses are difficult, or even impossible, to adapt to. This matters because adaptation reduces suffering. Illness severity is a priority setting criterion in several countries. When considering severity, we are interested in the extent to which an illness makes a person worse-off. I argue that no plausible theory of well-being can disregard suffering when determining to what extent someone is worse-off in terms of health. We should accept, all else equal, that adapting to an illness makes the illness less severe by reducing suffering. Accepting a pluralist theory of well-being allows us to accept my argument, while still making room for the possibility that adaptation is sometimes, all things considered, bad. Finally, I argue that we should conceptualize adaptability as a feature of illness, and thereby account for adaptation on a group level for the purposes of priority setting.

“… if adaptation to certain conditions takes place, this raises the difficult but unavoidable question if resource allocation decisions should take this into account as decision-makers may choose to prioritize conditions for which adaptation is less likely achieved.” (de Hond et al. 2019).

“I believe … that the hedonistic conception of happiness and especially of suffering, has enormous moral significance in its own right. It is what constitutes much if not most of the moral horror in experiences such as torture, starvation, military combat, disease, humiliation, clinical depression, and psychosis.” (Mayerfeld 1996).

The phenomenon of adaptation to illness has received a fair amount of interest in the literature on health care priority setting. This is partially due to the debate on whether health state valuations should be based on patient valuations or hypothetical valuations by the public, and the skepticism towards priority setting on the basis on adapted valuations which to an extent fuels this debate. In this paper I intend to investigate an aspect of adaptation that seems so far to have been mostly overlooked: that adaptation reduces suffering, and that some health conditions are difficult, or even impossible, to adapt to. When allocating resources in health care, several countries rely on considerations of the severity of illness,^1^ or other measures of who is worse off health-wise. Illness severity is influenced by many factors, one of which plausibly is the amount of suffering caused by the condition in question. Adaptation has the effect of reducing suffering, and suffering is a relevant factor on any reasonable theory of well-being relevant for health. If we disregard the effects of adaptation the result is a relative underestimation of the severity of conditions where adaptation is unlikely, resulting in a ranking of the severity of illness that does not accurately track the effects health conditions have on well-being.

The main objective of this paper is to make the argument that adaptation should count when considering to what extent someone is severely ill. Severity functions as a concept with both descriptive and normative features; it is both a description of an illness and it functions as a marker for priority. As such I will attempt to answer two questions: (1) does adaptation lead to a reduction in suffering that leaves a patient better off? And (2) is this reduction in suffering relevant when allocating healthcare resources? It is possible that severity, rather than being sensitive to worse off-ness per se, is sensitive to worse off-ness relevant for healthcare priority setting. Jølstad and Juth (2022) have recently considered, and rejected, such a relevance claim regarding the relationship between severity and worse-off-ness as a function of small differences in age, but perhaps the suffering associated with non-adaptation should be considered irrelevant. As regards to (1) I will argue that the reduction in suffering associated with adaptation is good, but that we should accept that adaptation can still be bad for a person, all things considered, due to factors other than suffering. I argue that the best way to preserve this possibility is to accept a theory of well-being that includes both objective and subjective factors. As regards to (2) I will argue that the suffering and lack off suffering associated with adaptation and non-adaptation is relevant for illness severity, and that adaptation can, and should, be assessed on a group level. Accordingly, adaptability is a feature of an illness that is relevant for severity and priority setting in health. The claim is not that adaptation should be a separate priority setting criterion, but that in assessing illness severity we must be sensitive to the effects of adaptation.^2^

I begin by introducing the notions of illness severity, health states, suffering, and adaptation to illness. The concept of adaptive preference is then discussed briefly. I then argue that adaptation makes an illness less severe in at least one sense: it reduces suffering, and this is relevant on any plausible theory of well-being. I then address the question of whether, and how, this reduction in suffering is relevant for distributional justice.

## Illness severity

When allocating health care resources, we typically wish to get as much health as possible, but it also matters to whom benefits accrue. Severity, or similar concepts such as need, is therefore used as a priority setting criterion in several countries, including the Netherlands, Norway and Sweden (Barra et al. 2020). The National Institute for Health and Care Excellence in the UK has also recently decided to adopt a severity modifier (National Institute for Health and Care Excellence 2022). In Norway, for instance, severity is a priority setting criterion alongside cost and benefit of interventions, with illness severity being operationalized as absolute (QALY) shortfall: the severity of a health condition is a function of how many good life years are lost compared to a reference life (Barra et al. 2020). The Norwegians and others who prioritize based on severity are willing to spend more resources *for the same benefit* to a severely ill person than to a less severely ill person. The importance of severity as a priority setting criterion is supported by the intuition that being badly- or worse off is morally important (Barra et al. 2020). Egalitarian, prioritarian and sufficientarian theories of distributive justice are all sensitive to the extent that someone is either badly- or worse off (Hirose 2014). A number of factors, including pain, disability, anxiety, loss of life years, reduced social functioning, age of the patient, risk of death, and others, could plausibly be claimed to contribute to illness severity (Barra et al. 2020; Jølstad and Juth 2022).

For the purposes of this article, I will assume two things about illness severity. The first is that illness severity is a descriptor of how bad the health condition is for a particular patient.^3^ A patient suffering from a severe health condition is, ceteris paribus, worse off than if their health condition was less severe, and vice versa. Secondly, I assume that severity constitutes a pro tanto reason to prioritize a patient for treatment. Patients suffering from a severe health condition should, ceteris paribus, be prioritized relative to patients suffering from a less severe health condition.^4^ Given these assumptions about severity my argument is relevant for the broader questions of *who is worse off when it comes to health and whom we should aid*. I develop the argument specifically in the context of illness severity for two reasons: first, as already mentioned, severity is used as a priority setting criterion in several countries. Answering the question of who is most severely ill is therefore an important practical question. Secondly, severity is a concept with intuitive moral significance in health care priority setting. Those with severe health conditions seem to merit our concern.

## Illness and suffering

Neither human ailment nor suffering are straightforward to define. One way of conceptualizing human ailment is to operate with the concepts of disease, illness, and sickness. In this classification, disease refers to an organic phenomenon, illness to the subjective aspects of a health condition, and sickness to the social aspects of health problems (Hofmann 2002). I intend to talk about something that includes all the above, using the terms illness and health condition as synonyms. While using “illness” in this sense is not aligned with some conceptions in the literature (e.g., Hoffman’s use), I find other generic terms (e.g., malady) somewhat awkward and hope that the reader will not be confused. I will not take a stance on how we should define illness as an ontological category, but my argument presupposes that the illness itself and the experience and impact of the illness can to some extent come apart. This seems to be a prerequisite for speaking about adaptation in the first place. There is thus the illness itself, with its associated or defining properties, and the experience and impact of the illness for a patient, with adaptation to at least some extent mediating the relationship between the two. One feature of an illness is the degree to which it is possible to adapt to it.

Suffering is something we know when we experience it, but defining it is not trivial. As is clear from the at the start of the article, Jamie Mayerfeld (1996) relies on a hedonistic account of suffering in his discussion, where suffering is understood in terms of negative experiential states. According to Bjørn Hofmann (2015), definitions of human suffering can be grouped according to whether they focus on threats to human agency, profound losses that impair life, or experienced negative sensations. While these forms of suffering might be interesting, I will follow Mayerfeld and focus on hedonic suffering in this paper.

## Adaptation to illness

The idea of measuring the value of different health states has been the subject of much scrutiny and criticism, especially by advocates of a mere-difference views on disabilities (Barnes 2009). A crucial point in this debate, and the essential point for the purposes of the argument presented here, is that the majority fails to understand the perspective of people living with disability. When considering life with a disability non-disabled people tend to imagine that life would be miserable. Research on the well-being of people with disabilities largely negates this view. People with disabilities report levels of happiness close to those of non-disabled people (Albrecht and Devlieger 1999). More generally, there tends to be a discrepancy between how the public and patients value health states, with patients typically valuing their health states more highly than healthy people value the same health states hypothetically (Damschroder et al. 2005). One reason for this seems to be that healthy people underestimate the level of adaptation (Ubel et al. 2005). Adaptation involves changing oneself in response to new circumstances (Menzel et al. 2002). This highlights that adaptation is adaptation to something, some *new circumstance.* The new circumstance remains unchanged during adaptation, whereas the persons attitude, experience, or handling of the circumstance changes. Menzel et al. (2002) list the following forms of adaptation: cognitive denial of dysfunction, suppressed recognition of full health, skill enhancement, activity adjustment, substantive goal adjustment, altered conception of health, lowered expectations, and heightened stoicism. Hedonic adaptation is also plausibly a factor in adaptation to illness (Mitchell 2018). Assuming that illness severity is a function of how bad a health condition is for a person, the public tends to view certain health conditions that patients adapt to as more severe than do patients suffering from these conditions.

Issues regarding this overestimation of the severity of conditions that patients adapt to has primarily been explored in the context of disability discrimination, especially through the notions of “double jeopardy” and “the QALY trap”,^5^,^6^ In this paper I focus on the converse aspect of this issue: namely the problem of non-adaptation. Some conditions are difficult, or maybe even impossible, to adapt to. Chronic pain is for example known to have a substantial and long-term effect on well-being (Daniel Kahneman and Krueger 2006). Patients suffering from depression value their health states lower than the general public (Pyne et al. 2009). It seems plausible to argue that these conditions are more severe than what the public thinks, and that this is at least partially because they have the feature of being difficult to adapt to.^7^

## Adaptive preferences

Before delving into the relationship between adaptation and suffering, I wish to briefly consider the concept of adaptive preferences. There is an obvious affinity between adaptation to illness and the notion of adaptive preference formation, and this matters because adaptive preferences are generally considered problematic for the purposes of distributive justice. Adaptive preferences have been the subject of extensive debate in the literature on ethics, just distribution, and rationality during recent years. Although intuitions on the matter are far from homogenous, there seems to be some agreement that adaptive preferences are “…shaped … by facts of, or perceptions of, availability or possibility.” (Dorsey 2017). Put simply, adaptive preference formation is a process where what you want is influenced by what you get. Adaptive preferences seem most problematic when they are shaped by unfortunate circumstances, especially if these circumstances can be avoided by human action, such as oppression. Disregarding adaptive preferences has been argued for on the basis of their irrationality (Eftekhari 2021), their undermining autonomy (Colburn 2011; Elster 1982) and the effects that accounting for them would have as regards the just distribution of resources (Sen 2009). It is common in the literature for the term not only to refer to preferences proper, but also to ‘… desires, values, commitments, and some beliefs and features of a person’s character.’ (Terlazzo 2017). Polly Mitchell (2018) has argued that adaptation to illness is not, for the most part, an instance of adaptive preference. She argues for taking patient valuations influenced by adaptation into consideration based on their not being adaptive preferences and because we should be careful not to engage in “denial of testimony”. My argument, though it engages with questions like the questions raised in the literature on adaptive preference formation, is neutral as to whether adaptation to illness involves adaptive preference formation. If they are, this does not necessarily undermine my arguments. To what extent we should disregard adaptive preferences is itself a matter of debate. Jessica Begon (2020) has recently defended a distinction between “well-being adapted preferences” that align with classic conceptions of adaptive preferences, and “justice adapted preferences”, which are ‘… preferences that are a poor guide to individuals’ entitlements.’. Some preferences might be adaptive in the first sense while being non-problematic for the purposes of the second, and vice versa. It is thus not clear that the questions of how a preference is formed and of whether a preference is relevant for resource claims come to the same things. So, even if problematic instances of adaptation are cases of adaptive preference, this does not necessarily justify dismissing the suffering entailed by non-adaptation.

## Does adaptation make you better off?

The first argument I wish to develop is that adaptation is relevant for severity by making people better off. This involves a series of claims. The first is that adaptation reduces suffering, leaving patients, ceteris paribus, better off. This claim has previously been defended by Torbjörn Tännsjö (Tännsjö 2019). The second claim is that disregarding adaptation amounts to disregarding non-adaptation, and that this means disregarding suffering. The third is that no plausible theory of well-being relevant for health can disregard the importance of suffering. Together with the assumption that severity is (at least partly) a function of worse off-ness, these claims support the conclusion that adaptation, ceteris paribus, makes a health condition less severe.

## Adaptation and suffering

One of the strongest reasons to be skeptical about accepting adaptation is that people adapt to circumstances they *should not* have to adapt to. Amartya Sen (2009) has forcefully argued this point regarding adaptive preference formation in response to poverty and deprivation. If all we consider relevant when determining how we should distribute is the happiness or preference satisfaction of individuals, then how are we to make a distinction between a poor person who is miserable due to lack of basic goods and a rich person who is, subjectively, equally miserable due to a lack of champagne and caviar? This is a compelling general argument against combining a pure subjective theory of well-being with a welfarist theory of justice. There is surely something to be said for not accounting for adaptation, for the sake of distributive justice. Consider the large number of destitute persons today. It is a tragedy that so many people are deprived of a proper education, adequate nutrition, and basic freedoms. It seems that this is cause for moral concern, regardless of the level of subjective well-being experienced by these people. But regardless of whether adaptation, or adaptive preferences, should be accounted for when distributing goods, it clearly seems to matter when considering someone’s level of well-being. Consider two people: person *A* is poor but happy (i.e., has a high level of subjective well-being). Person *B* is poor and miserable. Who is worse off? I think that most people will agree that it is preferable to be poor and happy rather than poor and miserable.^8^ In fact, this seems to be a clear case of one alternative dominating the other.

Sen’s argument against adaptive preferences implies that not discounting for the effects of adaptive preference formation, i.e., including adaptation, will result in unfair distribution. A similar point has been made regarding “bad” forms of adaptation. Menzel et al. (2002) argue that some forms of adaptation, most notably suppressed recognition of full health, cognitive denial, and lowered expectations, should not be considered when distributing resources.^9^ It seems intuitive to exclude the effects of these forms of adaptation, because they are instances of poor reasoning or irrational acceptance of poor (or irrational denial of good) circumstances.^10^ There seems to be two issues here: the familiar issue that including adaptation leads to an unfair distribution, and the intuition that some of these forms of adaptation are not good for people, all things considered. Some cases of adaptation, such as skill enhancement and goal adjustments, seem better than others, such as denial, for the people concerned. But while the argument that accounting for adaptation will lead to unfair distribution seems plausible, the argument that adapting does not increase someone’s well-being is more suspect: there is of course something bad about people having to resort to suppressed recognition of full health, cognitive denial, or lowered expectations, to endure their situation, but the situation would be even worse if these forms of adaptation did not reduce suffering. Even though we would prefer a world where people did not have to adapt to illness, there is undoubtedly one good thing to be said for adaptation, namely that it reduces suffering. Note that this is purposefully a weak claim. Rosa Terlazzo (2017, 2022) has made the stronger claim that objects of adaptive preferences can start out being bad for us, but become good due to personal development over time. I might, for example, change my valuation of what a good life consists in in response to mobility issues. If we accept this claim, the case for counting adaptation is even stronger.

Discounting adaptation, “bad” or not, amounts to a disregard of a difference in suffering when considering who is worst off. This, I believe, is inherently implausible. But surely some cases of adaptation are bad for people, all things considered, even if suffering is reduced. I will return to this question towards the end of the section and argue that accepting that well-being has both subjective and objective components allow us to accept the subjective benefits of adaptation, while leaving room for the possibility that some forms of adaptation are, all things considered, bad for people.

## Non-adaptation and suffering

Research suggests that adaptation to illness is highly domain-specific; some illnesses are easier to adapt to than others (Greene et al. 2016). Likely candidates for *non-adaptable health conditions* are certain forms of mental illness and chronic pain conditions. While not the only conditions that seem to resist adaptation (cancer and stroke are also difficult to adapt to), mental health conditions greatly impact subjective well-being and have empirically been found difficult to adapt to (Binder and Coad 2013). Chronic pain also seems to be difficult to adapt to (Greene et al. 2016), and has a substantial and lasting effect on subjective well-being (Daniel Kahneman and Krueger 2006). There are several theories as to why people do not adapt to these conditions: Binder and Coad (2013) suspect that the uncertainty involved in mental illness and pain is important, whereas Daniel Kahneman (2008) argues that adapting to depression and pain is difficult because “… the normal process of withdrawing attention from a steady situation is prevented.” Pain and various forms of mental illness force us to attend to them, making it hard to move on. It even seems plausible to argue, given the inherently subjective nature of the illness, that adapting to depression is a contradiction in terms. If disregarding non-adaptation implies that we underestimate the severity of illnesses such as depression, the result is a distributive scheme that fails to prioritize people with these conditions to the extent that their life is worse.

So, does disregarding adaptation imply disregard of non-adaptation? I will present two arguments for this, one analytic and one practical.

The most straightforward argument seems to be this: if one views adaptation as a good thing (and I have argued that even “bad” forms of adaptation include some goodness in the form of reduced suffering) then it is reasonable to view non-adaptation as a bad thing, all else equal. If we deem adaptation valuable because of its effect of reducing suffering, then not adapting is a disvalue. Returning to a person who requires a wheelchair after an accident, many of us would consider this a tragic, or at least a significantly bad, state of affairs. If this was a friend, we would wish that this person was able to lead a life of meaning and enjoyment notwithstanding their poor luck. We would clearly not be indifferent as to whether they managed this or not. We would find joy in the relief of suffering found by acquiring new skills or a changed perspective, and pity them if they were unable to accomplish this, resulting in more suffering. If one is indifferent to whether people adapt to their conditions, then it seems that one is forced to be indifferent as to the suffering caused by non-adaptation. In the context of considering to what extent someone is severely ill, or more broadly worse off because of their health, this seems unreasonable.

The second argument regards the relative severity of different health conditions, and the implications that these rankings of severity have for distributive matters. Priority setting is essentially a matter of relative distribution: some get more, some get less, and some get nothing at all. In health care systems such as the Dutch, Norwegian, or Swedish, who is prioritized is partly determined by who is most severely ill (Barra et al. 2020). When an illness is considered less severe, it will, all else equal, be outranked by illnesses considered more severe. Overestimating the severity of illnesses that patients typically adapt to means, relatively speaking, underestimating the severity of illnesses that patients do not typically adapt to. There is no neutral point when it comes to illness adaptation. Disregarding adaptation leads to disregarding non-adaptation in our rankings of illness severity.

## Theories of well-being

It might be objected that I have implicitly been assuming a hedonist theory of well-being in the previous arguments. On a hedonist account peoples well-being is defined by their subjective experience. My argument requires that subjective well-being, or more precisely the degree to which someone is suffering, is a necessary component of any theory of well-being relevant for health. I will now consider whether the argument holds on a desire satisfaction theory or objective theory of well-being. Regarding the desire theories the crucial question is what it means for a desire to be “informed”. Regarding objective theories the central question is whether a plausible theory of well-being can do without a subjective well-being component. I first turn to desire satisfaction theories**.**

### Desire satisfaction theory

Desire satisfaction, or preferentialist, theories claim that what a person’s well-being consists of is the satisfaction of their desires or preferences (Heathwood 2015). Much of the plausibility of these theories comes from the intuition that for *X* to be good or bad for a person, that person must care about *X*. As James Griffin (1986) has argued: Caviar might be considered great eating, but if I do not like caviar, then arguing that feeding it to me makes me better off is difficult. What is bad, on most forms of desire satisfaction theory, is frustrated desires. How to handle suffering on this account is not straightforward, because suffering and frustration do not seem synonymous. For the purposes of this paper, I will assume that a person is badly off from suffering because they have a frustrated desire not to suffer.^11^ For the purposes of this paper the main issue is to what extent a desire must be informed for it to contribute to a person’s well-being.

Griffin (1986) argues that any plausible desire satisfaction basis for welfare will demand that desires are to some extent informed. If my argument succeeds there are important goods associated even with “uninformed” preferences. Note that preferences can be uninformed in two ways: they can be faulty, and they can be formed by a dubious process. There seem to be two relevant points to be made when considering to what extent adapted preferences are informed:

1. Patients have a phenomenological closeness to their health states.
2. Adapted desires might be the result of cognitive distortions, irrationality, denial, or similar factors.

Considering the first point, Versteegh and Brouwer (2016) make an interesting argument: the public (and patients before becoming ill) do not know how it is to have a certain disease, but patients do not have direct phenomenological access to how it is to be healthy either.^12^ This argument is most plausible when considering patients who have never been in close to full health. However, many patients, in contrast to the public, have experienced both good health and the health states they are asked to evaluate. It thus seems likely that when considering tradeoffs between their health states and full health, these latter patients usually have a phenomenological edge when considering to what extent they are badly off. This leaves us with the question of cognitive distortions. Perhaps patients are in denial of their health or have shifted their expectations. At least, we cannot assume that this is never the case. Then, perhaps, the satisfaction of the desires that are based on these distortions should be considered less valuable. However, this is not the end of it. At this point we are entering familiar terrain. Whether or not patients have adapted in “bad” ways, surely their reduced suffering matters for their well-being. Perhaps patients are cognitively distorted when it comes to their health, but they are not plausibly completely misguided as to their subjective level of suffering, and we can assume that most people have a desire not to suffer.

### Objective theories of well-being

Objective theories of well-being claim that at least some factors influence well-being regardless of whether people subjectively value them or not (Parfit 1984). Prominent theories include various forms of perfectionism and objective list-theories.

On perfectionist theories of well-being, what matters for well-being is to what extent someone either has, develops, or makes use of essentially human capacities (Bradford 2015). Bradford (2017) has argued that perfectionisms failure to deal with the importance of pleasure makes perfectionism less plausible as a theory of well-being. Importantly, this only holds for perfectionist theories that do not include a subjective component. Perhaps the capacity to be happy is a human capacity that can be had or developed to various extents? In that case perfectionism could make sense of the relevance of suffering. Unless such a factor is included in a perfectionist theory, I agree with Bradfords point and add to this that a failure to deal adequately with suffering would also make perfectionism an unreasonable theory of well-being.

Objective list accounts of well-being generally have two features in common: they are attitude independent, in the sense that whether *X* is good for a person can be independent on that person’s attitude to *X*, and they are pluralistic. The second feature is not embraced by all theorists (some view hedonism as an objective list with one item) but the first is universally endorsed (Fletcher 2015). Attributes of certain illnesses might, on an objective list account, be bad for a person regardless of their attitude or experience; they may be attitude independent. Perhaps being blind, or having a mobility issue, is *intrinsically worse* than not, even if you do not value these things. If this is the case, there are factors making persons worse off health-wise that cannot in themselves be improved by adaptation. Importantly though, there seems to be a subjective component associated with these objective factors that is clearly improvable by adaptation. In fact, the concept of adaptation seems to require such a component. The previously mentioned candidates for non-adaptable health conditions, depression and chronic pain, are defined by their subjective experience, leaving less room for adaptation. The question then becomes whether it is plausible to assume that any account of well-being relevant for health can be indifferent as to subjective experience, and in particular suffering? An objective list without experienced well-being on the list seems implausible. If one where to come up with a list of factors that make your life go badly, suffering seems like an obvious contender. This is reflected in the works of philosophers working with an objective account. In Derek Parfit’s (2011) development of a value based objective account of reasons, pain is used as a paradigmatic case of an objective reason. T. M. Scanlon (2000), in his arguments against consequentialism, still acknowledges that we have a prima facie reason to prevent or avoid causing pain. On Daniel Hausman’s (2015) theory of the value of health, which can arguably be considered an objective theory, suffering/distress is one of two factors alongside functioning/limitations. This is not to claim that these authors accept a subjective theory of well-being, but simply to illustrate that it generally seems implausible to claim that you are not in some important sense worse off suffering than not suffering.

## Returning to “bad” adaptation

I believe that I have made the case that there is a positive side even to bad forms of adaptation, and that this goodness should be taken into account when considering the severity of illness. But this still leaves the question of whether all forms of adaptation are good, all things considered. The primary argument against counting adaptation, or adaptive preferences, seems to be the sour grapes argument. This is, as I argued previously, essentially an argument against combining a subjective account of well-being with a welfarist theory of distributive justice. Surely people’s objective circumstances matter. The argument seems to apply both to desire satisfaction and hedonist theories of well-being. The problem is, essentially, that there are important objective factors that the subjective theories fail to consider. I argued that it is worse to poor and miserable than poor and happy. This seems to be true, to the extent of being undeniable. But on a pure subjective account, the poverty involved can potentially loose its importance. In the same way, the fact that some forms of adaptation, such as denial or reduced expectation, are problematic, might be overlooked. On a pure subjective theory, we seem unable to differentiate between “good” and “bad” forms of adaptation in a way that aligns with our intuitions. In this regard an objective theory of well-being seems better poised to help us deal with the problem, provided it includes a measure of subjective well-being. Let us consider a new case of adaptation to illness: *A* has a massive amount of scarring to visible areas of their body because of a botched surgery. *A* previously had an active social and professional life, doing things that were very much worth doing. In response to the surgical scarring *A* becomes a recluse, spending their days indoors playing video games. After some time, *A* is happy spending their days gaming, and no longer has the wish to have an active social and professional life. Taking my argument seriously, there is obviously something good about *A* being happy doing what they are doing. It would clearly be worse if they were miserable. But at the same time is seems relevant that *A* no longer has an active life, doing things of substance and importance. On an objective theory we would be able to say that the adaptation is good in the sense that *A* is happy, but bad in the sense that they have given up things that are independently valuable. We could then weigh these factors when considering whether the adaptation makes the illness less severe. If we believe that the badness outweighs the goodness, the adaptation in question is bad, all things considered. This solution runs into the problem that we perhaps fail to show *A* what Rosa Terlazzo (2014) calls secondary recognition respect: we fail to recognize *A* as an authority on their own good. Any objective theory will plausibly run into this problem. While I am sympathetic to the idea that we owe people something like secondary recognition respect, I follow Serene Khader (2012) in rejecting the idea that ‘…it is disrespectful to treat another as though she has *some* bad values.’. As Khader reminds us, being subject to criticism is part of what it is to be a moral agent. On my account there are aspects of a persons’ well-being that we cannot plausibly criticize, namely their subjective experience. Any increase or decrease in subjective well-being should plausibly lead us to reassess the severity of a patient’s condition. Other aspects, the objective features, must be subject to debate and deliberation.

In assessing the overall severity of an illness or health condition, we must then weigh subjective and objective factors. While more needs to be said of such a theory, it would allow us to make sense of our intuitions in situations where subjective and objective features of well-being come apart or collide. Consider the following case reported by Mendez and Parand (2020), where a man presented with various cognitive impairments and a markedly joyful mood after a gunshot to the head. This was particularly striking because he had previously been disposed to suicidal ideation and depression. They write: “His mood appeared excessively good for the context, and his affect was congruent with mood but not overtly euphoric. He did not seem to be aware of the severity of his memory impairment, but he was aware that he had significant cognitive problems.” (Mendez and Parand 2020). Both a purely subjective and a purely objective theory of the value of health would miss out on important aspects relevant for well-being in this case. The pattern of a seeming disconnect between objective and subjective factors can be found in more common health conditions. While many people with Down’s syndrome struggle with various physical and cognitive problems, nearly 99% report being happy with their lives (Skotko et al. 2011). Cass Sunstein (2008) uses the case of Down’s Syndrome to illustrate how legal damages can be justified based on capability losses even when subjective well-being is unaffected. Similarly, a mixed theory of the value of health allows us to recognize that people with downs syndrome have high levels of subjective well-being, while not disregarding their medical and social needs in the process of allocating health care resources. So, as well as enabling us to make sense of the good and bad of adaptation, a theory including both objective and subjective factors give us the right result when making sense of our intuitions in other cases where subjective well-being and other aspects of health diverge.^13^

## Should adaptation influence healthcare priority setting?

So, I have established that adaptation can lead to a reduction in suffering that is relevant for assessing a person’s well-being, making it clear that adaptation is relevant for the worse off aspect of severity. I will now consider the relevance of this reduction in suffering for healthcare priority setting, and thus for the priority aspect of severity. I will argue that we should focus on adaptability as a feature of an illness. If some illnesses are easier to adapt to, leading to less suffering, this is relevant for distributional concerns. As mentioned in the introduction, the argument is not that adaptability should be a separate criterion for priority setting, but rather that it is a feature that we should account for when assessing the severity of illness.

Recall that any theory of well-being relevant to matters of health that does not give at least some weight to suffering is deeply implausible. In fact, not being able to deal with subjective suffering seems to make a theory something else than a theory of well-being as commonly understood. On any plausible consequentialist theory based on well-being relevant for health, suffering should be part of our ethical calculus.^14^ Allowing suffering to be overridden by other factors seem fine (if we accept an objective theory or are pluralists about well-being, it might often be the most reasonable option) but claiming that health related worse off-ness is not affected at all by whether a patient suffers seem unreasonable. We should therefore take illness adaptation into account when considering illness severity and when distributing resources, or so the argument goes. This can be problematic due to distributional considerations like those raised by Sen (2009), particularly if we restrict ourselves to a purely subjective theory of well-being. Adopting a pluralist theory of well-being might allay some of these worries, by letting factors other than suffering, such as basic flourishing, play a major part in our judgments of severity. We would then be able to consider the suffering involved in illness, while also being sensitive to other concerns.

So why should we focus on adaptability as a feature of illness? Prioritizing based on individual adaptation would clearly be a contested issue, largely because of practical concerns, but perhaps also due to considerations of desert or responsibility. The distinction between adaptability of illness and individual adaptation can isolate an argument that is likely to be less contested. Prioritizing based on features of illnesses, such as pain, reduction in life span, or loss of functioning, is an essential and non-controversial aspect of health care priority setting. On the practical side, assessing the extent to which illnesses are adaptable seems like a tractable problem, whereas assessing individual adaptation looks more problematic.^15^ On the question of desert or responsibility, the lack of adaptation to severe pain or depression does not seem to be as much a feature of particular people as of the illnesses in question. We are not plausibly responsible for features of our illness, making objections based on responsibility or desert mute. This argument should thus be plausible even to those who do not consider all forms of subjective well-being relevant for distributional concerns. Letting adaptability as a feature of illness influence our judgments of severity will result in sensitivity to the possibility of adaptation, while avoiding the problems associated with individual assessments. Accepting this argument might lead to prioritizing some conditions, such as depression and chronic pain conditions, to a larger extent that what we are currently doing. I mentioned in the introduction that the question of adaptation is important partly due to the controversy on whether we should use experienced or hypothetical measures when valuing health states. Another consequence of accepting the relevance of adaptability for severity is that adaptation is an argument *in favor* of including measures of experienced health when valuing health states for the purposes of priority setting. This is because the reduction in suffering achieved by adaptation is most effectively accounted for by asking patients to value their own health states. If we accept a pluralist or objective list conception of well-being, the most reasonable way forward is perhaps to include both measures of experienced health *and* measures of objective factors.

## Conclusion

I have argued that suffering makes a person worse off on any plausible theory of well-being relevant for health. Given that severity is at least partly determined by to what extent an illness makes a person worse off, judgements of the degree of severity must be sensitive to suffering. Non-adaptation to illness means that a person suffers more than if she had adapted to her illness. Unless our judgements of illness severity are to be insensitive to suffering, we are thus compelled, all else equal, to consider illness that one adapts to less severe and illness that one does not adapt to more severe. If we wish to claim that some forms of adaptation are bad for people, the most promising option seems to be to accept an objective theory with a subjective component. On the distributional question, I have argued that there is a strong case for being sensitive to the adaptability of illness when making judgments of severity, at least on a group level.

## Footnotes

1 I use ‘severity of illness’, ‘illness severity’ and ‘severity’ interchangeably through the article.

2 I would like to thank an anonymous reviewer for making me aware that my argument could be read as arguing that adaptation should be a separate priority setting criterion.

3 It may be a function of other parameters as well, but this is not anything I take a stand on here.

4 Although there may be other criteria for priority setting, for example cost and benefit of intervention.

5 «Double jeopardy” refers to the fact that people suffering from illness are, due to their lives containing fewer QALYs, less cost-effective to save when lives are at stake (Singer et al. 1995).

6 The QALY trap is described in the following quote: "If we want to value interventions that raise people’s quality of life, we are forced to give less value to saving the lives of those with lower quality of life. On the other hand, if we want to give equal value to saving the lives of those with lower quality of life, we are forced to give no value to raising people’s quality of life” (John, Millum, and Wasserman 2017).

7 Adaptation is separate from coping, which can be defined as “…the thoughts and behaviors mobilized to manage internal and external stressful situations. It is a term used distinctively for conscious and voluntary mobilization of acts…” (Algorani and Gupta 2022). While adaptation is a mostly relevant for long term illness, coping mechanisms are also important when dealing with illness in the short term. By focusing on adaptation, I do not intend to make any claims about the relevance of duration for the severity of illness. I focus on adaptation because it is a point of contention in the literature, where there are conflicting intuitions and theoretical commitment. While I do not discuss coping (denoting to short term illness), I believe that my arguments could be relevant for coping, in the sense that illnesses that are hard to cope with are extra burdensome. I thank an anonymous reviewer for bringing the distinction between coping and adaptation to my attention.

8 But for a contrary argument see Rickard (1995), who contends that adapting to degrading circumstances itself adds to the degradation, and therefore makes a person worse off.

9 They are unsure as to whether heightened stoicism makes a case for or against adaptation.

10 The question of the rationality or irrationality of adaptive preferences is complicated, especially because the process of character planning does not seem problematic or irrational to the same extent as other adaptive preferences (Elster 1982). If I change my preferences in response to my circumstances, there seems to be something rational about it to the extent that it improves my well-being. If I, on the other hand, kept my adaptive preference in the face of a change in circumstances, this might seem more irrational.

11 This seems somewhat forced. Bengt Brülde (2007) argues that what is bad on a desire theory is *aversion satisfaction*. This would make it easier to handle factors such as pain and suffering on a desire theory.

12 Strictly speaking they frame their point in the wording of ex-ante and ex-post valuations, given that valuation exercises involve non-experienced health states.

13 I believe that Daniel Hausman’s (2015) previously mentioned theory of the value of health could allow us to make sense of our intuitions on adaptation and illnesses without losses in subjective well-being, precisely because it includes both subjective (suffering/distress) and objective (functioning/limitations) factors.

14 If we would like to distribute in a manner that does not take adaptation into account, while retaining theoretical consistency, perhaps the way to go is to focus on means for living well rather than on well-being per se. This focus on means rather than ends is shared by Rawls theory of justice (Rawls 1999) and Nusbaum and Sens capability approaches (Nussbaum 2011; Sen 2009). I will not consider this here, other than to note that any consequentialist theory focusing on a balance between the maximization of and fair distribution of well-being, will be susceptible to my arguments.

15 Assessing adaptation on a group level would plausibly require studies on how the quality of life of patients, including health related quality of life and subjective well-being, change over time. These studies could be quantitative, such as Binder and Coad’s (2013) study on differing illnesses, but could presumably also include qualitative and mixed-methods studies. One way of accounting for the effects of adaptation could also be to include measures of experienced health in health valuations, since patient valuations avoid the underestimation of adaptation associated with hypothetical health valuations.
